# Thermoelectric materials by using two-dimensional materials with negative correlation between electrical and thermal conductivity

**DOI:** 10.1038/ncomms12011

**Published:** 2016-06-21

**Authors:** Myoung-Jae Lee, Ji-Hoon Ahn, Ji Ho Sung, Hoseok Heo, Seong Gi Jeon, Woo Lee, Jae Yong Song, Ki-Ha Hong, Byeongdae Choi, Sung-Hoon Lee, Moon-Ho Jo

**Affiliations:** 1 Division of Nano and Energy Convergence Research, Daegu Gyeongbuk Institute of Science and Technology (DGIST), 333, Techno Jungang Daero, Hyeonpung-Myeon, Dalseong-Gun, Daegu 42988, Korea; 2 Center for Artificial Low Dimensional Electronic Systems, Institute for Basic Science (IBS), Pohang University of Science and Technology (POSTECH), Pohang 790-784, Korea; 3 Department of Electronic Material Engineering, Korea Maritime and Ocean University, Busan 49112, Korea; 4 Division of Advanced Materials Science, Pohang University of Science and Technology (POSTECH), Pohang 790-784, Korea; 5 Korea Advanced Institute of Science and Technology (KAIST), Daejeon 305-701, Korea; 6 Korea Research Institute of Standards and Science (KRISS), Daejeon 305-340, Korea; 7 Department of Materials Science and Engineering, Hanbat National University, Deajeon 34014, Korea; 8 Department of Materials Science and Engineering, Pohang University of Science and Technology (POSTECH), Pohang 790-784, Korea

## Abstract

In general, in thermoelectric materials the electrical conductivity *σ* and thermal conductivity *κ* are related and thus cannot be controlled independently. Previously, to maximize the thermoelectric figure of merit in state-of-the-art materials, differences in relative scaling between *σ* and *κ* as dimensions are reduced to approach the nanoscale were utilized. Here we present an approach to thermoelectric materials using tin disulfide, SnS_2_, nanosheets that demonstrated a negative correlation between *σ* and *κ*. In other words, as the thickness of SnS_2_ decreased, *σ* increased whereas *κ* decreased. This approach leads to a thermoelectric figure of merit increase to 0.13 at 300 K, a factor ∼1,000 times greater than previously reported bulk single-crystal SnS_2_. The Seebeck coefficient obtained for our two-dimensional SnS_2_ nanosheets was 34.7 mV K^−1^ for 16-nm-thick samples at 300 K.

## Abstract (web summary)

The improvement of the thermoelectric figure of merit ZT has been hindered by the challenges associated with the independent control of the electrical and thermal conductivity. Here the authors show that SnS_2_ nanosheets can lead to an increased ZT via negative correlation between electrical and thermal conductivity.

## Graphical abstract

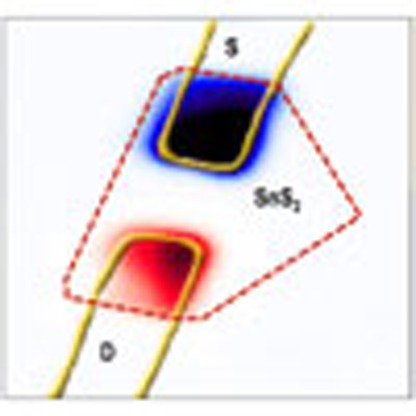

Waste heat has been the most significant loss of useful energy in heat engines since their discovery. Recovery of waste heat will enhance energy efficiency, reduce greenhouse emission and promote sustainable development. Thermoelectric (TE) devices have shown potential for reclaiming waste heat, whereas TE generators contain no mechanical parts and are therefore useful for long-term operation.

TE effect refers to a phenomena wherein a temperature difference across a material creates an electric potential, commonly called the Seebeck effect^1^,^2^,^3^,^4^,^5^, or the opposite case where electric potential creates a temperature difference, as seen in Peltier cells^1^,^6^. Since its discovery, researchers have struggled to improve the efficiency of the TE, represented by the dimensionless figure of merit, *ZT=*(*S*^*2*^*σ/κ*)*T*, where *S*, *σ* and *κ* are the Seebeck coefficient (also called TE power), the electrical conductivity and the thermal conductivity of the material, respectively. To improve *ZT* we have continued to design materials and structures that have an enhanced electrical conductivity *σ* and reduced thermal conductivity *κ*. Recent research into nanomaterials has led to increased *ZT* through nanomaterials or -structures and quantum confinement effects to obtain an enhanced density of states near the Fermi energy^2^,^3^,^4^,^5^,^6^,^7^,^8^,^9^,^10^,^11^.

As nanoscale structures have become known, their counterintuitive properties have intrigued experimental and theoretical workers. The benefits of nanoscale structures have been applied across various fields from fin-type field-effect transistor structures for semiconductors^12^ to drug delivery^13^ and targeting in medicine^14^. In view of TE materials, several different types of nanostructures such as Si nanowires (*ZT*_200 K_ 1)^15^, quantum nanodotAgPb_*m*_SbTe_2+*m*_ alloys (*ZT*_800K_ 2.2)^5^, Bi_2_Te_3_/Sb_2_Te_3_ superlattices (*ZT*_300K_ 2.4)^8^ and layered structure of two-dimensional (2D) crystalline sheets^16^,^17^ have been used to improve *ZT* by using low-dimensional structures. These general ideas have been used to greatly decrease the thermal conductivity, while suppressing the decrease in electrical conductivity as shown in Fig. 1. The progress of TE materials are described in Fig. 1, showing the trend of *ZT* for different materials classes.

More recently, with the discovery of the 2D carbon allotrope graphene^18^, a great deal of interest has been focused into 2D nanostructures^16^,^17^. Most importantly for TE materials, the electrical conductivity of layered 2D materials were in some cases greater than their bulk material counterparts^18^. Surprisingly, for nanoscale SnS_2_ we found that although the electrical conductivity increases in 2D structures, the thermal conductivity in fact decreases. This class of materials leads to negative correlation between enhanced electrical conductivity *σ* and reduced *κ* exactly as we would like for TE applications. SnS_2_ has a unique structural property of layered CdI_2_-type structure; the tin (Sn) atoms are sandwiched by two layers of hexagonally packed sulfide (S) atoms^19^. Each layer has a thickness of 6−7 Å. The intra-layer metal (M)-chalcogenide (X) bonds are predominantly covalent in nature, whereas the layers themselves are coupled by weak van der Waals^19^ bonds. The metal atoms provide four electrons to fill the bonding states of SnS_2_ such that the oxidation states of the M and X atoms are +4 and −2, respectively. The lone-pair electrons of the chalcogen atoms terminate the surfaces of the layers and the absence of dangling bonds renders those layers stable against reactions with environmental species. In addition, very recent theoretical work using first principle calculations in ref. 20 have suggested *ZT* values as high as 0.96 at room temperature, which shows potential for TE applications.

## Results

### Synthesis and investigations of TE properties of SnS_2_

We were able to succeesfully fabricate pure SnS_2_ single crystals by thermal chemical vapour transport (CVT). To our knowledge, this is first time CVT has been successfully used to fabricate SnS_2_ single crystals for electrical measurement (for further growth details and characterization of SnS_2_ nanosheets, see Methods and Supplementary Figs 1−4).

**Figure 2:**
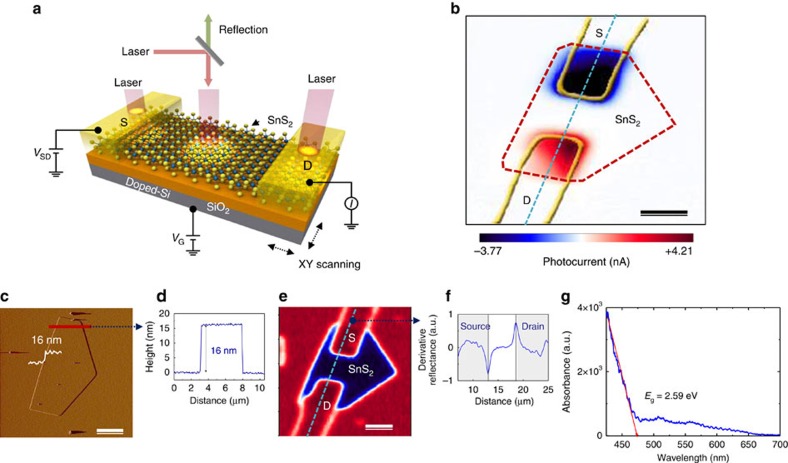
Thermal current measurement of SnS_2_ by laser scanning photoinduced TE current imaging. Devices are 16 nm thick. (**a**) Schematic of the photo-TE measurement setup with a laser wavelength of *λ*=405 nm and laser power of 45 μW, where we simultaneously collect images of photo-TE current and optical reflectance as a function of the laser position. (**b**) Scanning photo-TE current imaging (0 V source/drain bias), which demonstrates largely thermocurrent-dominated profile in the source (S)/drain (D) electrode regions. The outline of the electrodes (yellow solid lines) and the SnS_2_ nanosheet (red dashed line) are indicated from reflection image of **e**. Scale bar, 3 μm. (**c**) AFM image of SnS_2_. Scale bar, 5 μm. (**d**) Thickness measurement along the red line in the AFM image. (**e**) Reflection image. Scale bar, 3 μm. (**f**) Numerical derivative of the reflectance data along the blue dashed line allows for clear indication of S/D and SnS_2_ boundaries. (**g**) Ultraviolet–visible absorption spectroscopy. The absorption edge of SnS_2_ films showing the bandgap at 2.59 eV, extrapolated from the *x* intercept of the linear portion of our data (red line).

One method of investigating the TE effect in materials has been the use of focused laser to generate thermal current through local heating, creating a temperature gradient^16^,^17^. We investigated the total photocurrent in 16-nm-thick SnS_2_ sample by using laser wavelengths of 405 nm (3.06 eV). The beam spot size was 500 nm and scanned across the entire crystal, while measuring the current through grounded drain electrodes. The experimental setup and explanations are included in Fig. 2a. Figure 2b shows the photocurrent map (0 V source bias) under the laser power of 45 μW. The large current values appearing on the source/drain electrodes indicate the presence of high thermal current; in contrast, most materials show only current at the metal/semiconductor interface due to photoelectric currents^17^.

The thickness of our samples was measured by atomic force microscopy (AFM) and confirmed to be 16 nm as shown in Fig. 2c,d. Figure 2e shows the reflection image of Fig. 2b and numerical derivative (Fig. 2f) of the reflection signal, which allows for a clear indication of source/drain and SnS_2_ boundaries. Figure 2g shows ultraviolet–visible absorption spectroscopy. The absorption edge of SnS_2_ films for a 16-nm-sample show a bandgap (*E*_g_) of 2.59 eV. Meanwhile, the 100-nm-thick SnS_2_ film was measured to have a bandgap of 2.15 eV (for the dependence of optical bandgap on SnS_2_ thickness, see Supplementary Fig. 5).

**Figure 3:**
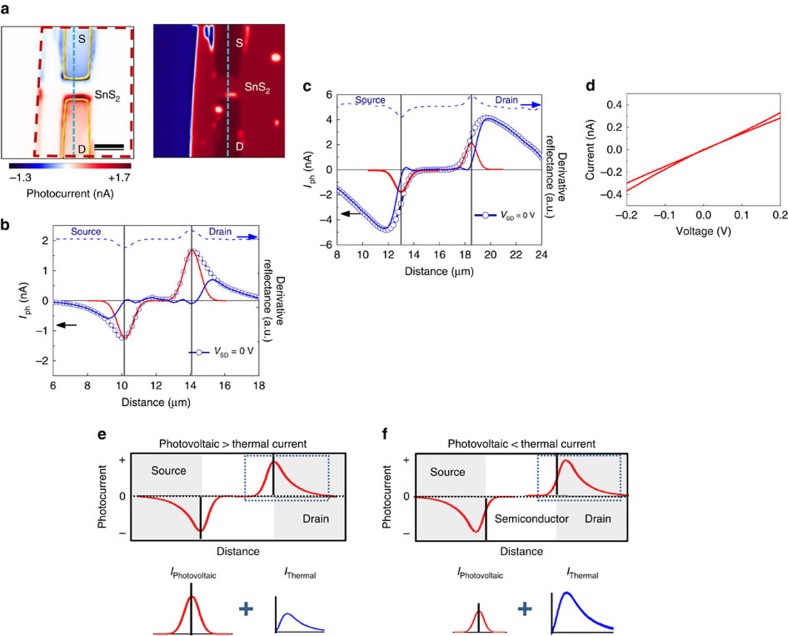
Photocurrent composition measurement and analysis for two SnS_2_ samples of thickness. (**a**) Photocurrent map (left) and reflection image (right). Scale bar, 3 μm. (**b**) Photocurrent profile at 0 V source bias with laser wavelength of 405 nm (3.06 eV) and power of 195 μW for 100 nm thickness, which shows photovoltaic current-dominated Gaussian profile. The derivative of reflectance along the blue dashed line shown in the reflectance measurements (right of **a**) plotted in the photocurrent profile. (**c**) Photo-TE current profile from along the blue dashed line in Fig. 2b, which demonstrates a thermocurrent-dominated profile. (**d**) *I*–*V* curve of the Pt/Ti/SnS_2_ contact, demonstrating that a good ohmic contact is formed on the SnS_2_ material. (**e**,**f**) Expected current profile and decomposition of contributing components for the 100- (**e**) and 16-nm (**f**) samples. Shaded areas indicate S/D electrode regions. The measured photocurrent in the dotted blue rectangles of **e** (top) and **f** (top) are decomposed into their respective Gaussian photovoltaic (bottom left) and thermal (bottom right) components; their sum results in the profile shown in each respective dotted blue rectangle region.

We also investigated the photocurrent in two different SnS_2_ samples of thickness 16 and 100 nm in the same way as previously shown in Fig. 2a. Figure 3a shows the photocurrent and reflectance images at 0 V source bias and Fig. 3b shows photocurrent profile of a 100-nm-thick SnS_2_ crystal illuminated with laser power of 195 μW laser. The large Gaussian current profile is not due to the photovoltaic effect, but a photo-TE effect, similar to previous reports^17^. However, we should note that the larger Gaussian current profile for 100 nm samples compared with 16 nm ones is probably due to the lowering of the bandgap, leading to a more pronounced photovoltaic effect in the thicker samples as shown in Supplementary Fig. 5. We also used a 532-nm (2.33 eV) laser on the same 100-nm sample and found that the current features are similar even though *hν*<*E*_g_ of SnS_2_ (see Supplementary Fig. 6). To clearly identity source/drain interfaces, we used the measured reflectance (right of Fig. 3a) and show the derivative (blue dotted line of fig. 3b). The local minimum and maximum of the blue dotted line indicate the position of the source/drain interfaces. The solid red and blue lines in the photocurrent profiles of Fig. 3b,c are the Gaussian components and the results after subtracting each Gaussian component from the experimental data, respectively.

Figure 3c shows photocurrent profile (0 V source/drain bias) of a 16-nm-thick SnS_2_ (2D layered structure) crystal illuminated with laser power of 45 μW. The photocurrent profile is extracted from the photocurrent image previously shown in Fig. 2b. The photo-TE profile was changed compared with the 100-nm sample as observed in Fig. 3b. Figure 3d shows current–voltage (*I–V*) characteristics (source–drain) of Ti/Au contact on SnS_2_ nanosheet exhibited ohmic behaviour. If the photocurrent is dominated by photo-TE current, we can expect a current profile as the one in the diagram of Fig. 3e. Here we notice a Gaussian profile reflecting the intensity profile of the laser beam spot. The Gaussian peak is centred at the metal–semiconductor interface where the internal field due to the Schottky barrier is maximized. Almost identical profiles have been previously reported for MoS_2_ materials where photo-TE effects dominate the photocurrent^17^. When the TE effect dominates the photocurrent, we expect a different distribution rather than the Gaussian profile, because the temperature gradient rather than the laser intensity affects carrier distribution. The distribution peak is also moved into the electrodes, rather than the interface, as explained by the diagram of Fig. 3f. These features are well in agreement with our observed photocurrent profiles.

The layered structure materials including SnS_2_ can be cleaved down to few or single layer, with significant changes to the electrical and optical properties such as indirect-to-direct bandgap transition^21^,^22^. The evolution of electronic properties of SnS_2_ nanosheets with various thicknesses can be reflected in their photo or thermal current. The bulk SnS_2_ showed mall thermal current, whereas thinner SnS_2_ nanosheets exhibited pronounced thermal current at 16 nm thickness (Figs 2b and 3b).

### Negative correlation between electrical and thermal conductivity

Our results suggest that the TE effect is the dominant photocurrent mechanism in layered 2D (16 nm) SnS_2_. We believe this is due to the unique properties of 2D SnS_2_ where the thermal conductivity decreases with decreasing thickness, due to surface phonon scattering, whereas at the same time electrical conductivity increases with decreasing thickness, possibly due to changes in the band structure similar to graphene^18^. By using this property it may be possible to maximize *ZT* beyond any previous approach. We have done an electron density change calculation by using Synopsys technology computer-aided design for SnS_2_, showing increased electron density at reduced thickness, which we present in Supplementary Fig. 7, Supplementary Note 1 and Methods.

**Figure 4:**
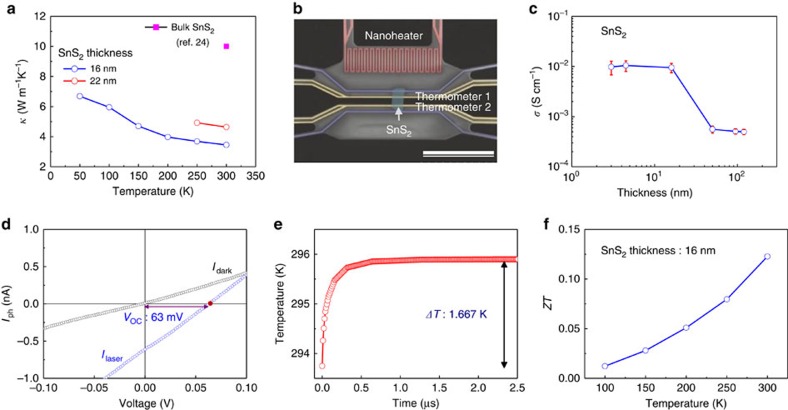
Negatively correlated electrical and thermal conductivity. (**a**) Thermal conductivity measurements for 16- and 22-nm-thick SnS_2_ samples compared with bulk SnS_2_ reported values. (**b**) Scanning electron microscopy image used for MTMP setup and precise thermal conductivity measurement. Scale bar, 30 μm. (**c**) Electrical conductivity measurements for different thickness of SnS_2_ showing a large increase below 16 nm. The median value of three samples is shown by open circles, whereas error bars indicate the maximum and minimum values from each sample. (**d**) Open circuit voltage (*V*_oc_=63 mV) measurement of 16-nm SnS_2_ sample under illumination by 405-nm laser (power of 45 μW). (**e**) Simulated *ΔT* for SnS_2_ samples illuminated by the same laser as left-hand side figure. (**f**) Calculated *ZT* values for temperatures from 100 to 300 K.

To further investigate the changes to thermal conductivity in 2D SnS_2_ crystal, we use the previously reported microfabricated TE measurement platform (MTMP) device, which was shown as a method to accurately measure Bi_2_Te_3_ thin film conductivities previously^23^. In brief, the MTMP method uses a differential method to exactly measure thermal conductivity of materials precisely by using a microelectromechanical system-based device^15^,^23^. A full description of the MTMP method is included in the Supplementary Figs 8 and 9. The results of the 16-nm film show a thermal conductivity of 3.45 W m^−1^ K^−1^ at room temperature; the temperature-dependent thermal conductivity is shown in Fig. 4a and plotted alongside is the reported value for bulk SnS_2_ in ref. 24 at 300 K. A separate 22-nm sample was also measured later, to confirm the thermal conductivity dependence (Supplementary Fig. 10), and included in Fig. 4a. In comparison with bulk, this is around one-third of the reported 10 W m^−1^ K^−1^ in ref. 24 at 300 K. Figure 4b shows scanning electron microscopy image of MTMP structure used for thermal conductivity measurements with current-supplying nanoheater and temperature-measuring thermometer metal leads. The sheet resistivity of SnS_2_ nanosheets was measured using a four-point Van-der Pauw method for samples from 120 to 3 nm thickness (for four-point Van-der Pauw method, see Supplementary Fig. 11). We multiplied the film thickness confirmed by AFM and took the inverse value to find electrical conductivity *σ*(*T*) in Fig. 4c. Between 16 and 50 nm of SnS_2_ thickness, there is a 30 times increase in the electrical conductivity from ∼ 4 × 10^−4^ to ∼ 1 × 10^−2^ S cm^−1^. We believe this region to describe the change from bulk three-dimensional properties to 2D properties for SnS_2_ thin films. Meanwhile, for bulk SnS_2_ electrical conductivity has been reported as 0.9 S cm^−1^ (ref. 25).

## Discussion

From *ZT=*(*S*^*2*^*σ/κ*)*T*, everything else being constant, the one-third decrease in *κ* and the 30 times increase in *σ* lead to a total *ZT* increase of over 90 times for 2D SnS_2_ in comparison with that of three-dimensional SnS_2_. Finally, the Seebeck coefficient was measured by using the open circuit voltage (*V*_oc_) of devices as shown in Fig. 4d, leading to *V*_oc_=63 mV for 16 nm devices (for the Seebeck coefficient for 150-nm SnS_2_ thickness, see Supplementary Fig. 12).

Using the definition of the Seebeck coefficient and simulated *ΔT* value of 1.667 K (Fig. 4e) for conditions exactly as described in Fig. 2b, we have |*S*|=26.1–34.7 mV K^−1^ (*ΔT* simulations along with calibration methods using Bi_2_Te_3_ described in Supplementary Figs 13 and 14, Supplementary Notes 2 and 3, and Supplementary Table 1), with an overestimation error of ∼28%. In comparison, the reported absolute values for bulk Sn_*x*_S_*y*_ are 0.1–4.6 mV K^−1^ in ref. 26 and measurement values for our 100-nm-thick SnS_2_ sample are around 3.2 mV K^−1^, with an overestimation error of ∼28%. We expect a large increase in *S* to enhance local electron density from confinement effects, similar to previously reported nanostructures^6^. Finally, we calculate *ZT* values (16 nm SnS_2_) from 0.012 at 100 K to 0.13 at 300 K from our measured values as shown in Fig. 4f, which are close to the highest values being reported for TE materials currently^3^,^4^,^5^,^6^. The measured electrical conductivity dependence on temperature for 16 and 100-nm samples is included in the Supplementary Fig. 15.

In summary, at this time the final *ZT* values was around other reported nanomaterial values of 0.13 at 300 K. We believe a significant increases can be made by theoretical screening for materials, which have negatively correlated electrical and thermal conductivity with a layered structure similar to SnS_2_, while having higher absolute electrical conductivity. Currently, researchers believe a room temperature *ZT* of 0.5 (a factor of 4 higher than this work), which could reach a *ZT* of over 3 at high temperature (900 K), to be useful for industrial applications^3^,^4^. By investigating materials that have negative correlation between *σ* and *κ*, we suggest that moderate-temperature TE materials can be discovered.

## Methods

### Synthesis and characterization of SnS_2_ nanosheets

Synthesis of SnS_2_ was carried out by thermal CVT in an evacuated tube furnace using 300-nm-thick SiO_2_ substrate. SnO_2_ (99%, nanopowder with particle size under 100 nm, Sigma-Aldrich) and sulfur powder (99%, Sigma-Aldrich) precursors were used for the synthesis of SnS_2_, with temperature range of 600–680 °C (Supplementary Fig. 1). Triangular faceting of single-crystal SnS_2_ was observed by optical microscopy. The crystal structure and their characterization was confirmed with transmission electron microscopy, Raman and absorption spectrum (see Supplementary Figs 2 and 3). The morphology and the number of layers are determined by Raman and AFM imaging.

### Electrical characterization of SnS_2_

SnS_2_ samples for laser and electrical measurement were fabricated by electron-beam lithography on the wafers that were previously doubly spun with two polymethyl methacrylate layers. Electrodes are evaporated in a high vacuum e-beam evaporator and are composed of 5 nm per 50 nm titanium (Ti)/gold (Au) layers. Subsequently, the polymethyl methacrylate/Ti/Au layer is lifted off in acetone. The basic electronic transport characterization of the SnS_2_ devices is performed at room temperature and atmosphere. The scanning photocurrent microscopy is carried out at room temperature in a confocal microscope setup with an objective with numerical aperture=0.8. The excitation was provided by a focused laser of a given wavelength (405 and 532 nm) and by a supercontinuum white-light source (Fianium Ltd) combined with a monochromator for the high-resolution spectra (450 nm≤*λ*≤2,000 nm) where *λ* is the wavelength. During the wavelength scanning, photocurrent is measured by a lock-in technique with the chopper frequency of 500 Hz and subsequently normalized to the photon flux. The chopped laser beam is focused by microscopic lens (numerical aperture=0.8) and illuminates the SnS_2_ channel and S/D electrode region of devices.

### Fabrication and measurement for the thermal conductivity

The thermal conductivity of SnS_2_ nanosheets was measured by using the MTMP based on the differential method first demonstrated in ref. 23. That is, to exactly measure the thermal conductivity, we used the difference of the heat flow between devices including SnS_2_ flakes and without SnS_2_ flakes (see Supplementary Figs 8−10). MTMP structures with current supplying and temperature measuring metal leads were fabricated on Si_3_N_4_ (50 nm thick)/300-nm-thick SiO_2_ substrates. The micro-sized electrode patterns (Pt nanoheater, current-supplying electrodes (outer electrodes) and Pt thermometers (inner electrodes)) were photolithographically defined as shown in Supplementary Fig. 8. For enhancing the measurement sensitivity, the device area between the inner electrodes was removed: first, by etching the Si_3_N_4_/SiO_2_ wafer front side with hydrogen fluoride (HF) solution, then aligning the backside photoresist patterns to expose the Si and etching with a 30% KOH etching solution at 353 K. The temperatures were obtained from the resultant responses of the Pt thermometers to electrical resistance variations. The temperature gradient, *ΔT*, was generated by the Joule heating using a DC current (Keithley, 6220) in the range 0−64 μA through the Pt nanoheater. Two lock-in amplifiers (Signal Recovery, 5210) simultaneously read the resistances of both thermometers. The resistances of the Pt thermometers were converted into temperature values using the temperature coefficient of resistance (TCR). We determined the TCR for each individual microelectromechanical systems device before conducting the TE measurement, as a small difference in the TCR would lead to significant deviation from the real value when reading the temperature. From 200 to 400 K, the resistances of the Pt thermometers, which changed linearly with the temperature, were measured at the interval of 20 K in the temperature range of 200–400 K. The TCRs were determined from the slope of resistance versus temperature.

### Calculation methods

Electron density profiles and normalized conductance data shown in Supplementary Fig. 7b,c are calculated by Synopsys technology computer-aided design, a commercial semiconductor device simulator in ref. 27. Poisson equations and drift-diffusion equations are selected for calculating electrostatic potential and carrier transport, respectively, which are represented by following equations:


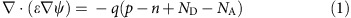



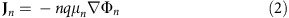



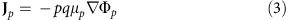


where *ɛ*, *ψ* and *q* are the electrical permittivity, the electrostatic potential and the elementary electronic charge, respectively; *n* and *p* are the electron and the hole densities, *N*_D_ and *N*_A_ are the concentration of ionized donors and acceptors, respectively; 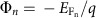 and 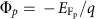 are the quasi-Fermi potentials for the electrons and holes, respectively; **J**_*n*_ and **J**_*p*_ are the electron current density and the hole current density, respectively. The generation of *n*-doped region by surface states of SnS_2_ is modelled by shallow donor-like interface trap formation. The schematic and the parameters for the device simulation are described in the Supplementary Fig. 7a and Table 1, respectively.

### Data availability

The data that support the findings of this study are available from the corresponding author upon request.

## Additional information

**How to cite this article:** Lee, M. -J. *et al*. Thermoelectric materials by using two-dimensional materials with negative correlation between electrical and thermal conductivity. *Nat. Commun.* 7:12011 doi: 10.1038/ncomms12011 (2016).

## Supplementary Material

Supplementary Information Supplementary Figures 1-15, Supplementary Table 1, Supplementary Notes 1-3 and Supplementary References

## Figures and Tables

**Figure 1:**
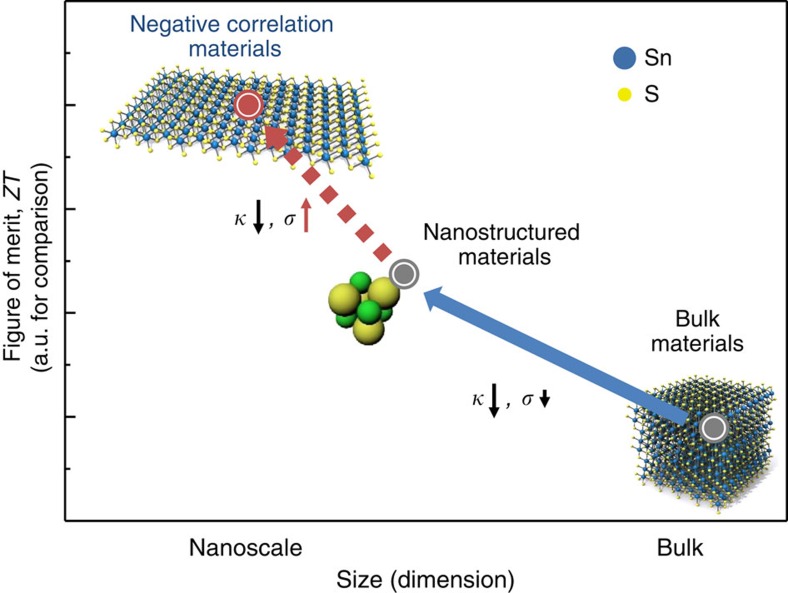
Pathway and progress for high *ZT* in TE materials. Conventional bulk materials have values from 0.01 to around 1, by using nanostructures, values between 0.1 to 2 have been reported for SnSe (*ZT*_300 K_ 0.12; ref. 4), quantum nanodot AgPb_*m*_SbTe_2+*m*_ alloys (*ZT*_800 K_ 2.2; ref. 5), Bi_2_Te_3_/Sb_2_Te_3_ superlattices (*ZT*_300 K_ 2.4; ref. 8) and Si nanowires (*ZT*_200 K_ 1; ref. 15). We present negatively correlated materials, which offer another method for increase of *ZT*. The solid blue arrow indicates previous progress, while the dotted red arrow is our proposed approach for further increased *ZT*.

**Table 1:** The parameters of SnS_2_ for the device simulation

Parameter	Value
Band gap	2.24 eV (ref. 28)
Dielectric constant	9 (ref. 29)
Electron affinity	7.30 eV (ref. 28)
Effective mass of electron	0.545 m_0_ (ref. 30)
Doping density of SnS_2_	10^12^ cm^−3^
Interface trap density	10^12^ cm^−2^
Energy level of interface trap	0.1 eV from cond. band
Electron mobility	22.5 cm^2^ V^−1^ s^−1^ (ref. 31)
