# Supplementary material for: Thermoelectric materials by using two-dimensional materials with negative correlation between electrical and thermal conductivity
Source: Nat Commun. 2016 Jun 21;7:12011. doi: 10.1038/ncomms12011 (PMC4919538; doi:10.1038/ncomms12011)
Supplement: Supplementary Information — Supplementary Figures 1-15, Supplementary Table 1, Supplementary Notes 1-3 and Supplementary References [file ncomms12011-s1.pdf]

Supplementary Figures

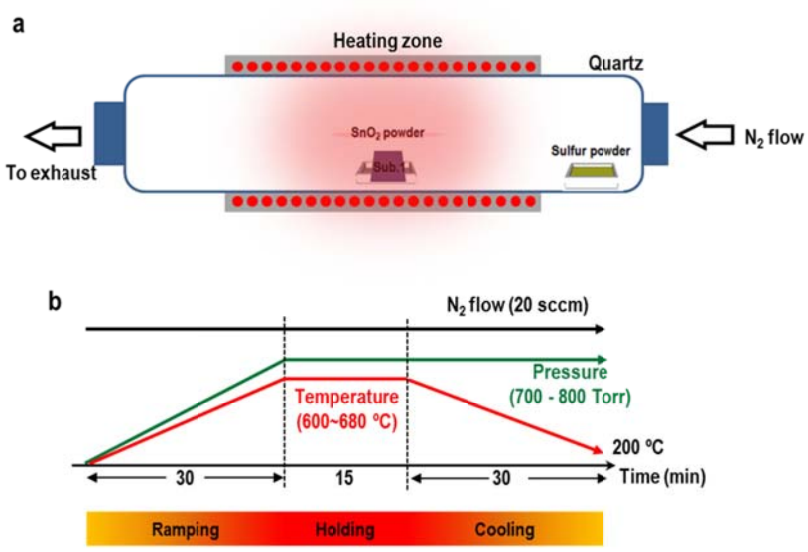

Supplementary Figure 1. (a) Schematic of thermal chemical vapor transport (CVT). (b) Synthesis procedure showing operating conditions: time, temperature, gas flow rate, and pressure for the synthesis of SnS_2 nanosheets. Two-dimensional nanosheets of SnS_2 were synthesized on SiO_2/Si substrates by a vapor transport method inside a 12-inch length hot wall quartz tube furnace. Tin (IV) oxide nanopowder 00 nm particle size, Sigma Aldrich) and pure sulfur powder were used as solid precursors and reactants. As shown in **Supplementary Fig. 1a**, the SnS_2 powders, loaded into the center of heating zone, were placed in an alumina boat and the SiO_2/Si growth substrate was faced down and mounted on the top of the boat. The sulfur powders, with the relatively lower melting temperature of about 115 °C, in a separate alumina boat was loaded outside the heating zone of 120 °C. Prior to our growth process, the furnace was evacuated to 10^{-3} torr and was purged by flowing the 400 sccm of high-purity N_2 during 10 min. Then, an inert ambient was established by flowing of N_2 20 sccm. The furnace temperature was gradually increased to the growth temperature of 600-680 °C in 30 min, and kept for 15 min for the growth. In the cooling step, the chamber was cooled to 200 °C with cooling rate of 15 °C per min and then rapidly cooled to room temperature. During the whole process, the chamber pressure was maintained at 700-800 Torr.

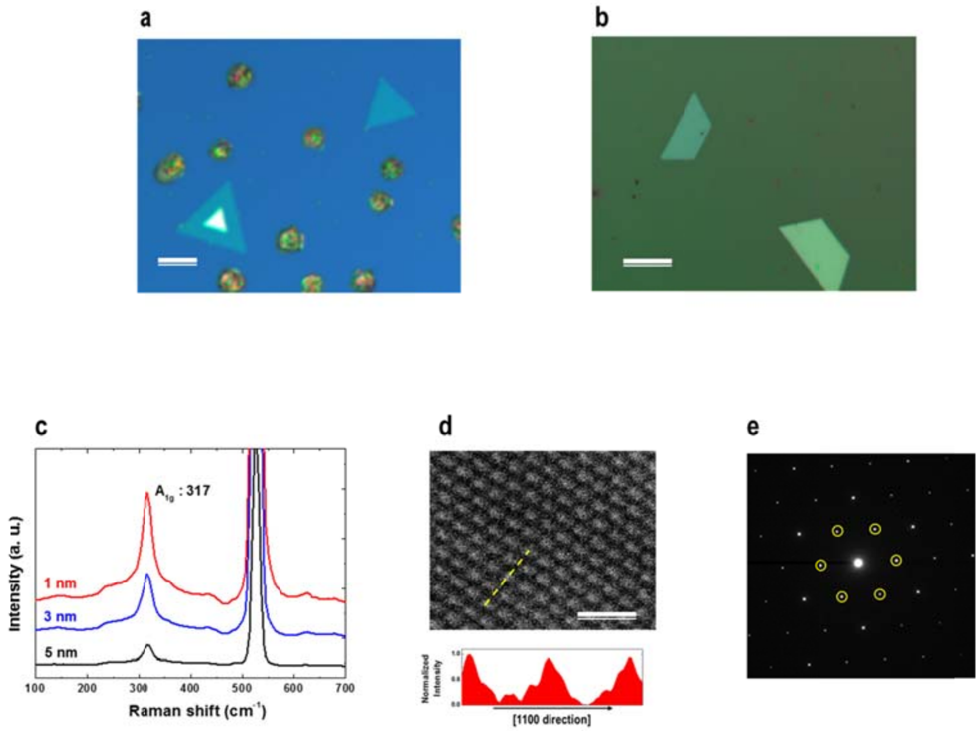

Supplementary Figure 2. Characterization of thermal chemical vapor transport (CVT) grown SnS_2 nanosheets. (**a**) Optical microscopy image with a range of 1~10 nm thickness well-faceted triangular SnS_2 nanosheets. Scale bar, 20 μm. (**b**), Optical microscopy image with a range of 15~100 nm thickness SnS_2. Scale bar, 20 μm. (**c**) few-layer SnS_2 has distinctive signatures in its Raman spectrum. 1 nm thickness is corresponding to the bi-layer. The main Raman peaks correspond to the out-of-plane A_{1g} mode at 317 cm^{-1}. (**d**) STEM-HAADF image demonstrating the defect-free hexagonal structure of the triangular SnS_2 single crystal in **a**. Intensity profile along the yellow dashed line indicated in image that was corresponding to the 1 T structure. Scale bar, 1 nm. (**e**) The FFT pattern for the SnS_2 layer demonstrating crystalline structure for SnS_2.

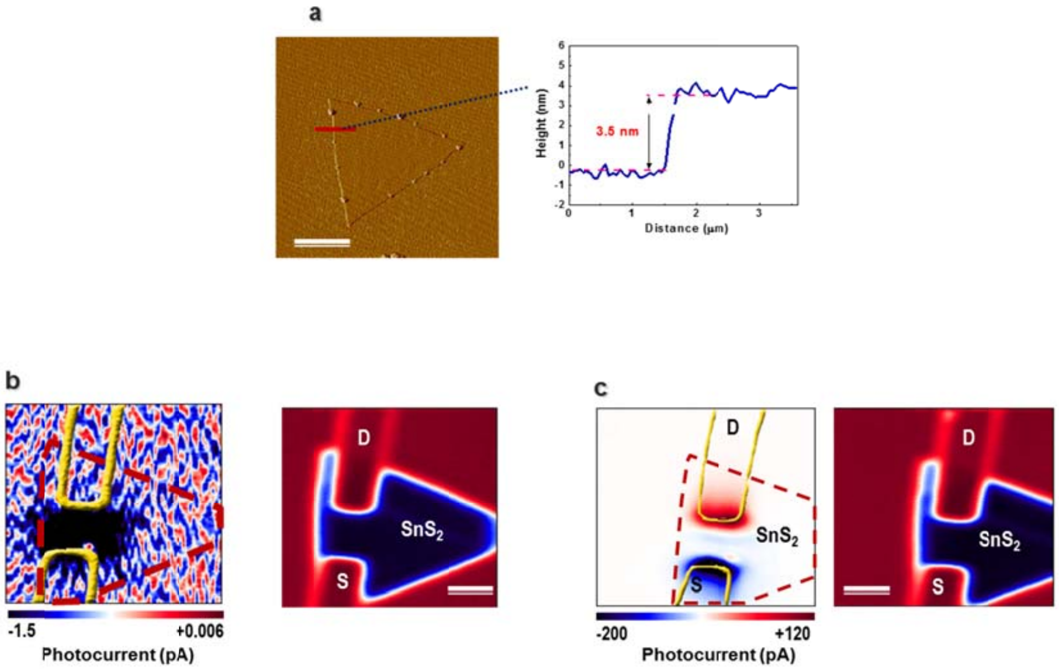

Supplementary Figure 3. Laser scanning photoinduced thermoelectric current imaging for 3.5 nm SnS_2 thickness. (**a**) AFM image of 3.5 nm thickness SnS_2 (left) and thickness measurement (right). Scale bar, 5 μm. (**b**) Photocurrent imaging and reflectance image with laser wavelength of 405 nm and power of 35 μW which demonstrate photo induced current dominated image in SnS_2 between source and drain electrodes. Scale bar, 2 μm. (**c**) Photocurrent imaging and reflectance image with laser wavelength of 405 nm and power of 130 μW. Photocurrent is dominant in S/D interfaces and electrodes region. Scale bar, 2 μm.

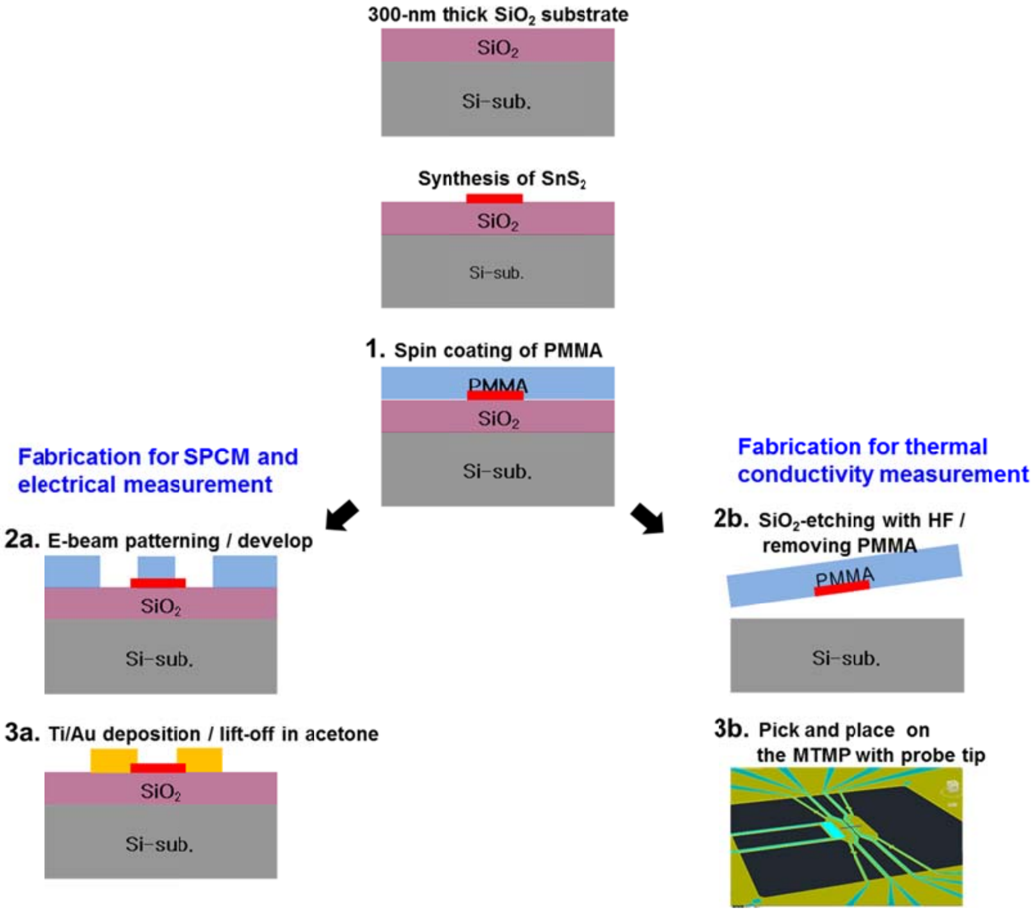

Supplementary Figure 4. The fabrication process flow of the SnS_2 devices for SPCM, electrical and the thermal conductivity measurements. The process illustrate the two different fabrication processes for SPCM and thermal conductivity measurements. For both SPCM and electrical measurement: first, PMMA resists was spin coated at 1000 rpm for 10 sec followed by 4000 rpm for 50 sec resulting in a 500 nm thickness PMMA film after a hot plate bake at 200 ℃ for 2 minute. Second, E-beam lithography was used to pattern electrode regions (**2a**). Third, Ti (5 nm) /Au (50 nm) was deposited using an evaporator followed by a lift-off process (**3a**). For thermal conductivity (MTMP) measurements: The first step was identical to SPCM samples. During the second step we removed the PMMA+SnS_2 layer by etching through SiO_2 with a 10 % HF solution (**2b**). Third we transfer the PMMA+SnS_2 onto previously prepared MTMP substrate by using probe tips with an applied bias (0.01 V) for electrostatic attraction. Finally, Pt contacts were formed using FIB deposition (**3b**).

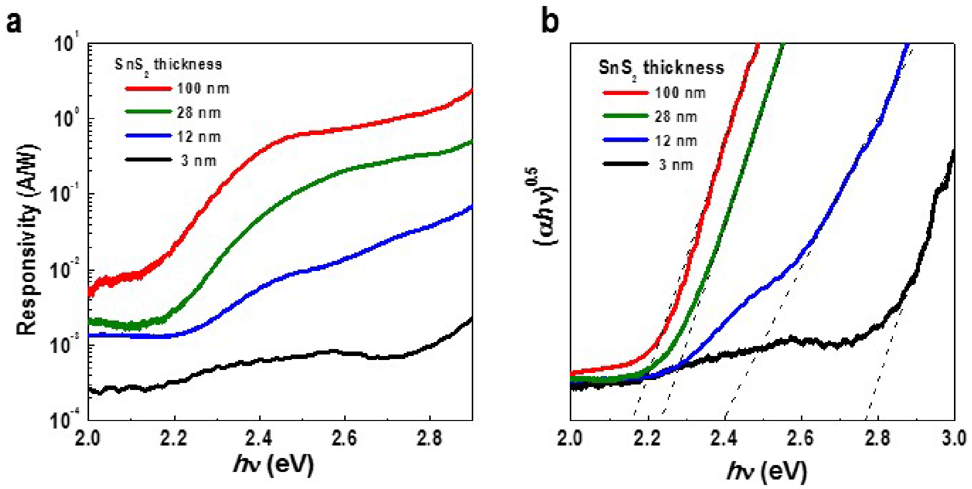

Supplementary Figure 5. Thickness dependence of (**a**) spectral responsivity ($I_{ph}/h\upsilon$) and (**b**) optical bandgap of SnS_2 crystal extracted by extrapolating the linear region of $(\alpha h\upsilon)^{1/2}$ vs $h\upsilon$ plot. The absorption edge of SnS_2 films showing the bandgap at 2.76 eV for 3 nm, 2.4 eV for 12 nm, 2.24 eV for 28 nm, and 2.15 eV for 100 nm respectively.

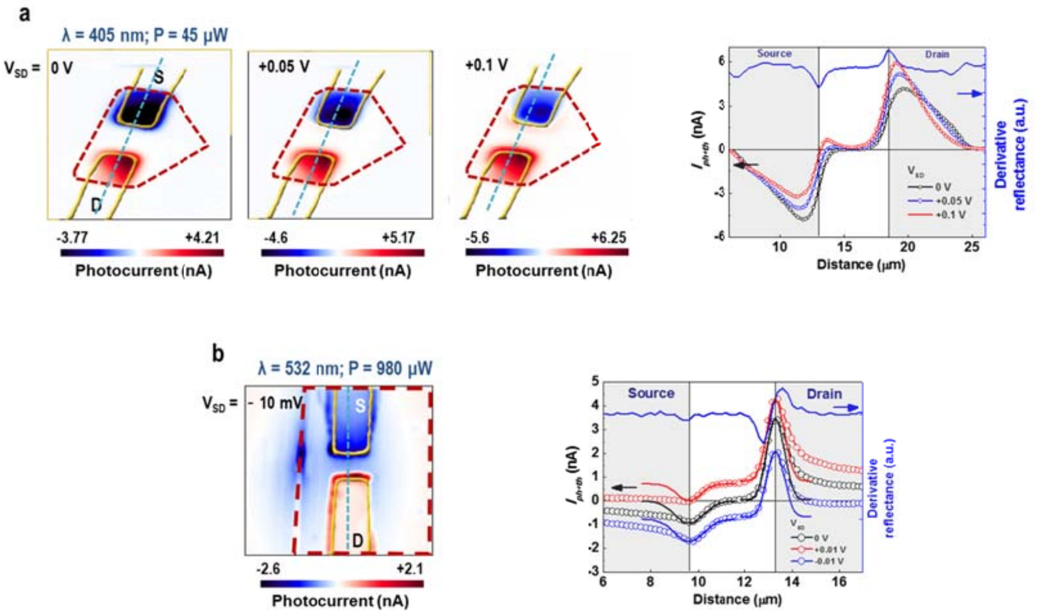

Supplementary Figure 6. Photocurrent composition measurement and analysis in different SnS_2 thickness. (**a**) Photocurrent map and profile with source-drain bias from 0 V to 0.1 V and laser wavelength of 405 nm (3.06 eV) and laser power of 45 μW. The bandgap of SnS_2 is measured to be approximately 2.59 eV leading to photovoltaically generated carriers at the source/drain interfaces, which can be distinguished by the moving in photovoltaic current directions with source biased conditions. (**b**) Photocurrent map and profile at 0 V with laser wavelength of 532 nm (2.33 eV). The current profile was similar to 405 nm even though hν (2.33 eV) < E_g of SnS_2. Therefore the current originates from photothermoelectric effect rather than photoelectric effect (electron-hole pair generation across the bandgap). Reversing bias does not affect photocurrent profile. The derivative of reflectance along the blue dotted line shown in the photocurrent map plotted in the photocurrent profile.

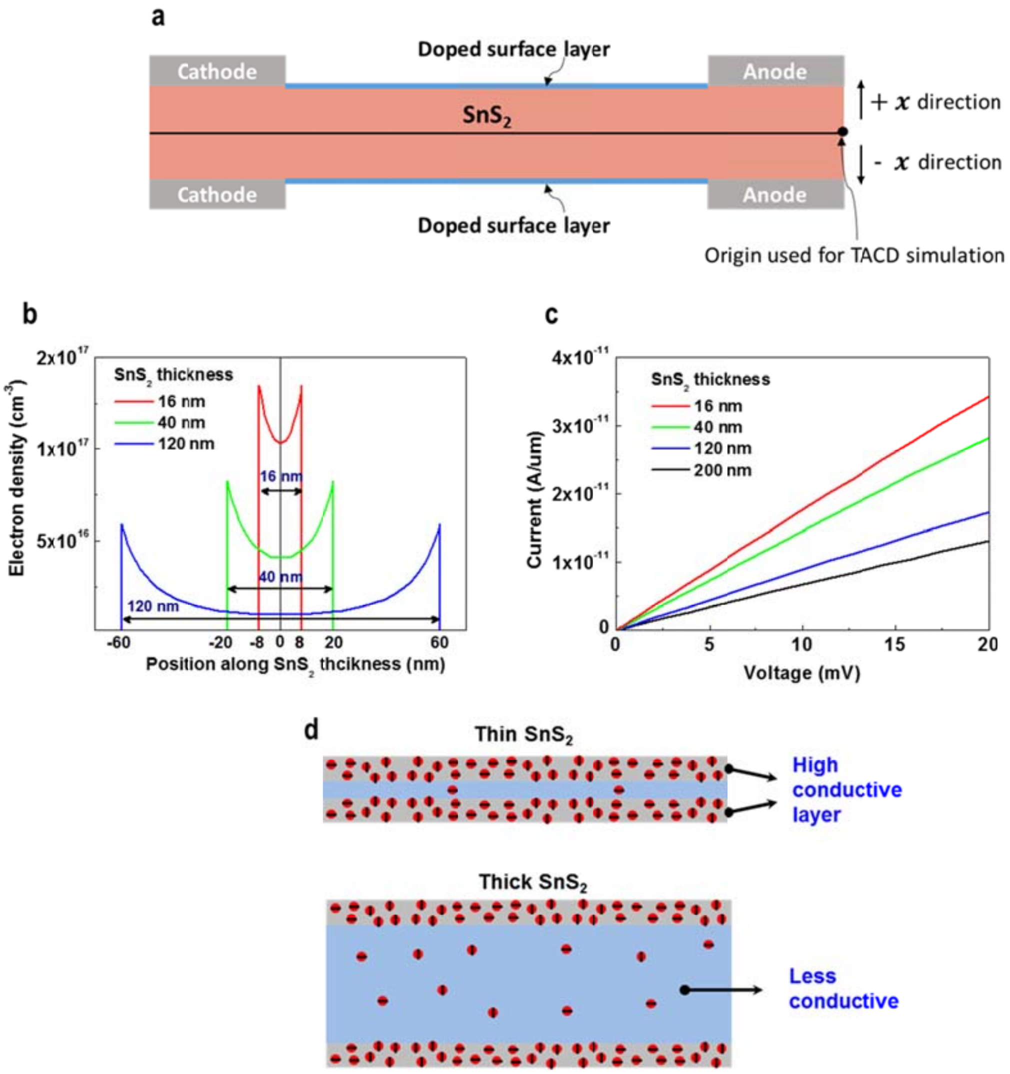

Supplementary Figure 7. Electron density simulation as SnS_2 thickness. (**a**) Schematic of device simulation. (**b**) Calculated electron density as a function of the SnS_2 thickness. (**c**) *I-V* as a function of the SnS_2 thickness. (**d**) Schematic of the thick and thin SnS_2 affected by surface doping. In the case of thin SnS_2, high conductive laver is dominated in the whole electrical conductivity.

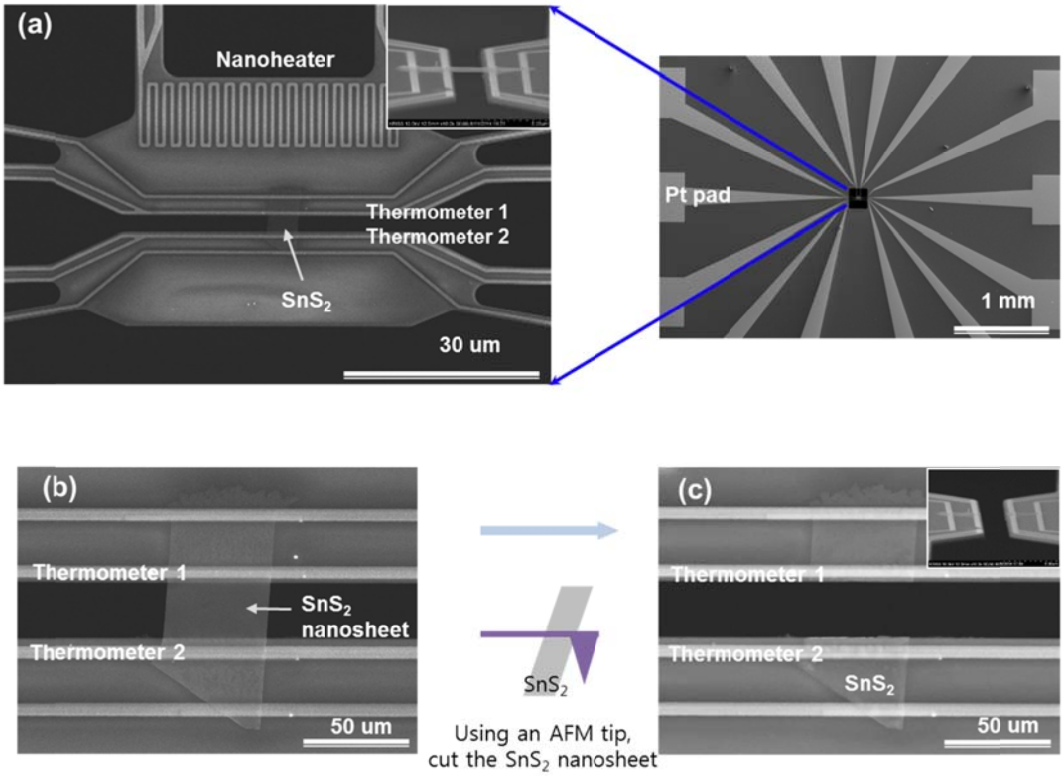

Supplementary Figure 8. SEM images of microfabricated thermoelectric measurement platform (MTMP). The figures show SEM images of before and after the SnS_2 nanosheet cut for definition of measurement of SnS_2 thermal conductivity. MTMP structures were fabricated on Si wafer, with silicon nitride isolation thickness: 500 nm, Pt electrode: width 500 nm, thick 40 nm, and an opening area: 200 μm × 200 μm. (**a**) MTMP structure used for thermal conductivity measurements with current supplying and temperature measuring metal leads. Inset is an enlarged image rotated 90 degrees and tilted. All metal lines including nanoheater are fabricated with Pt. (**b**) Magnified image of (a) before cutting the 16 nm SnS_2 nanosheet. (**c**) Magnified image of (a) after cutting the 16 nm SnS_2 nanosheet. Inset is an enlarged image rotated 90 degrees and tilted.

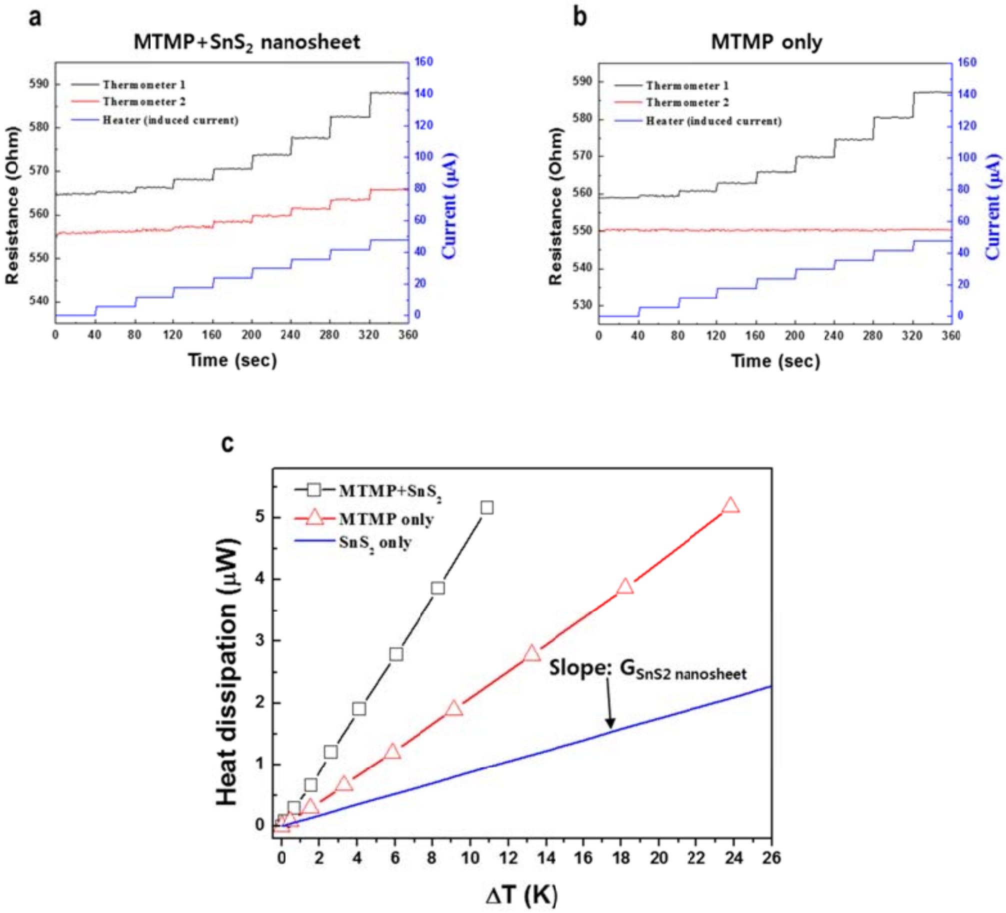

Supplementary Figure 9. Thermal conductivity measurement of SnS_2 nanosheets using MTMP structure. (**a**) Resistance measurement before cutting 16 nm SnS_2 nanosheet at thermometer 1 (black) and 2 (red). Blue line is the difference between the values at each thermometer. (**b**) Resistance measurement after cutting 16 nm SnS_2 nanosheet at thermometer 1 (black) and 2 (red). Blue line is the difference between the values at each thermometer. After cutting heat is not transferred to thermometer 2, leading to constant resistance value. (**c**) Calculated heat dissipation by the following:

The rate of heat flow *Q* through the wall is then given by $Q = k\frac{A}{L}\Delta T$, where *κ* is the thermal conductivity, A is the cross-sectional area of the wall normal to the direction of heat flow, L is the distance of T (T_1-T_2) , i.e. $\frac{dT}{dx} = \frac{T_1 - T_2}{L}$ Supplementary equation (1). If the Q measurement value without a SnS_2 nanosheet is $Q_{\text{MTMP only}}$ and the *Q* measurement value with a SnS_2 nanosheet is $Q_{\text{MTMP+SnS2}} = Q_{\text{MTMP only}} + Q_{\text{SnS2}}$, the

thermal conductance of SnS_2 is given by $\boldsymbol{G}_{MTMP+SnS2} = (Q_{MTMP+SnS2} - Q_{SMTNPonly}) / \Delta T$ Supplementary equation (2). Therefore, the thermal conductivity of SnS_2, k, is as follows: $k = (Q_{MTMP+SnS2} - Q_{SMTNPonly}) / \Delta T \bullet (L_{SnS2} / A_{SnS2})$ Supplementary equation (3).

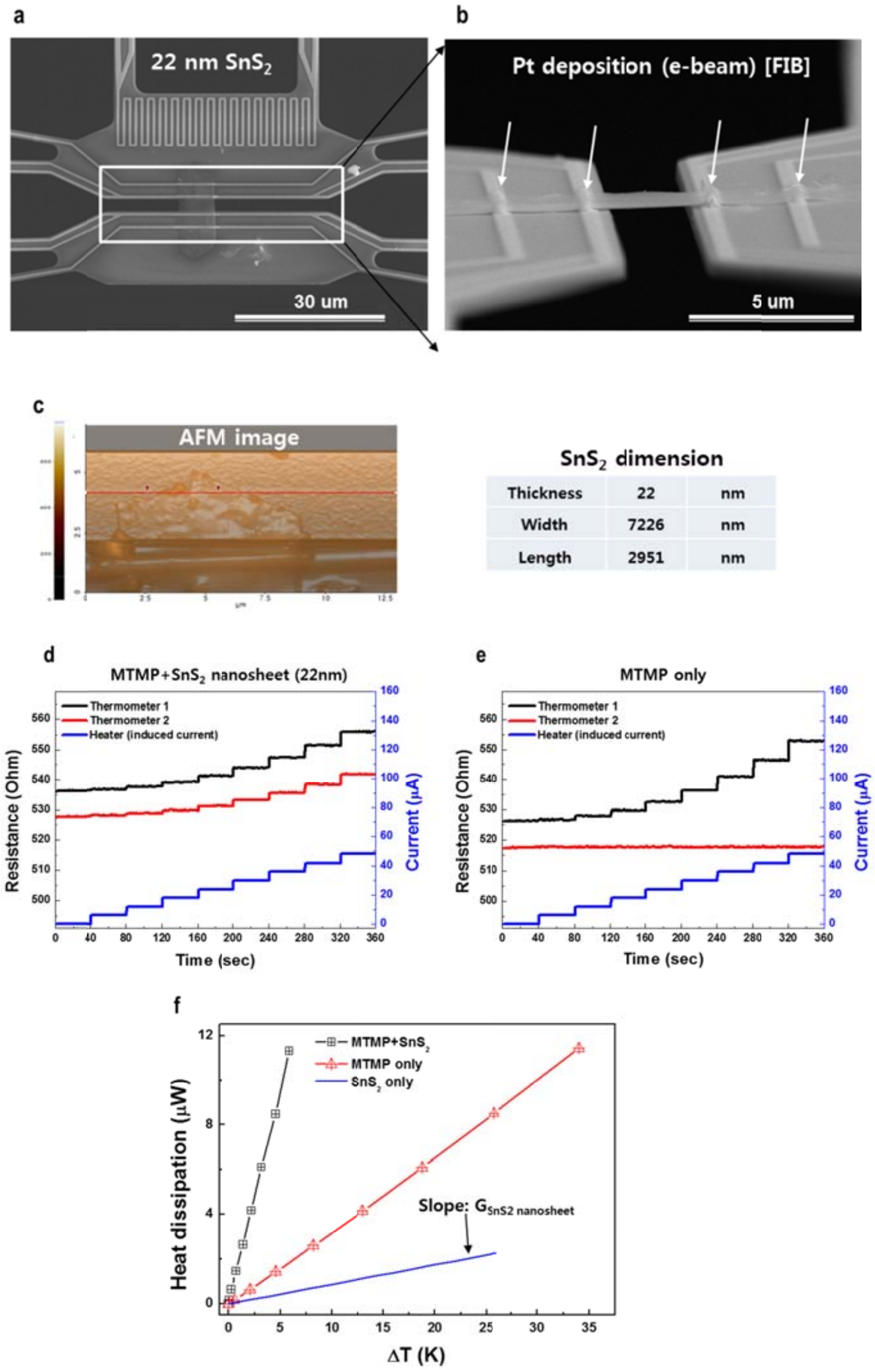

Supplementary Figure 10. (**a-c**), SEM and AFM image for 22 nm SnS_2 thickness using MTMP structure. (**d**) Resistance measurement before cutting 22 nm SnS_2 nanosheet. Blue line is the difference between the values at each thermometer. (**e**) Resistance measurement after cutting 22 nm SnS_2 nanosheet at thermometer 1 (black) and 2 (red). (**f**) Calculated heat dissipation of 4.63 W m^{-1} K^{-1} at 300 K.

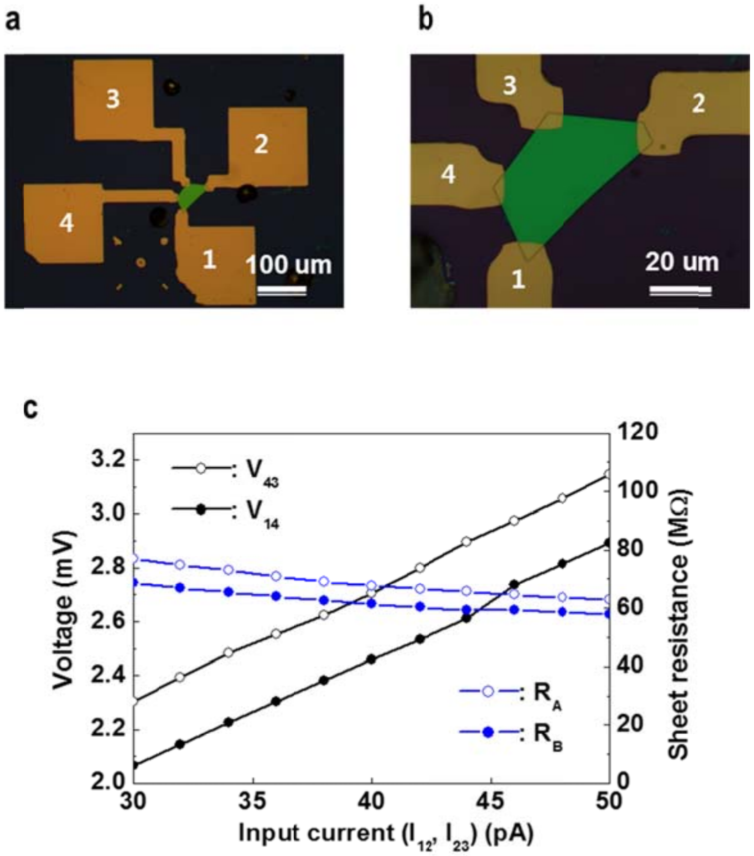

Supplementary Figure 11. Resistivity measurement of SnS_2 using the 4-probe Van der Pauw method. (**a**) Experimental setup image for SnS_2 electrical conductivity measurement by Van-der Pauw Method. 4 contacts are made near the sample edges for sample sizes ranging from 3 nm to 120 nm. (**b**) Magnified image showing contacted areas on SnS_2 film for 120 nm sample. (**c**) Measured values for V_{43} (open circles) and V_{14} (closed circles) when supplying current I_{12} and I_{23} respectively. Extracted sheet resistance values are shown on the right axis.

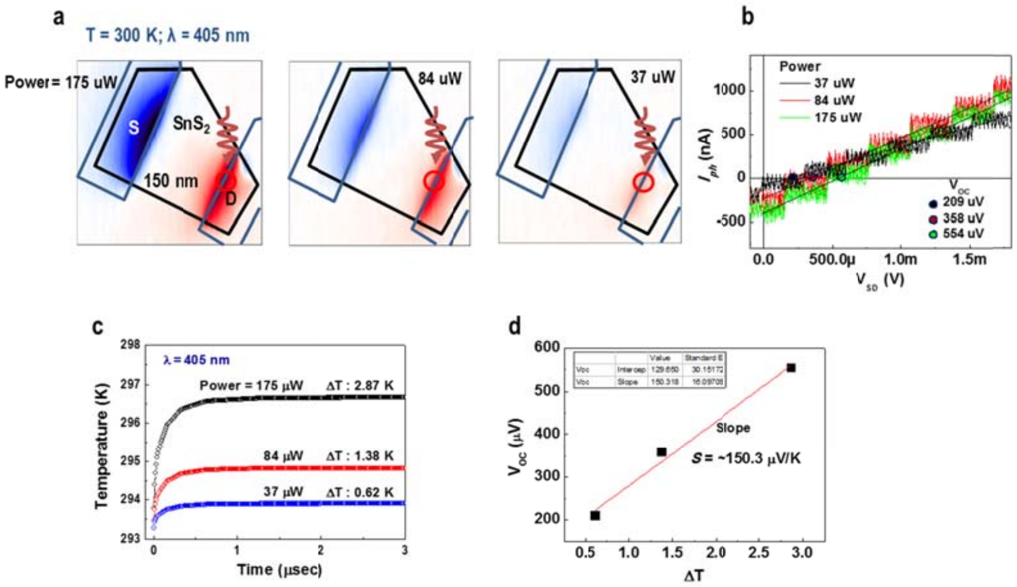

Supplementary Figure 12. Photovoltage composition measurement (at 300 K, 405 nm) and temperature difference simulation for Seebeck coefficient in 150 nm SnS_2 thickness. (**a**) Photovoltage map and I_{ph}-V as a function of laser power of 175, 84 and 37 μW. (**b**) I_{ph}-V of (**a**) while illuminating the drain side with laser. (**c**) Simulated ΔT saturation on the different laser power using modeled system as mentioned in **Supplementary Fig. 13**. (**d**) V_{oc} vs. ΔT plotted to measure the slope, Seebeck coefficient of 150 μV K^{-1} ($S = V_{oc}/\Delta T$) for 150 nm SnS_2 thickness.

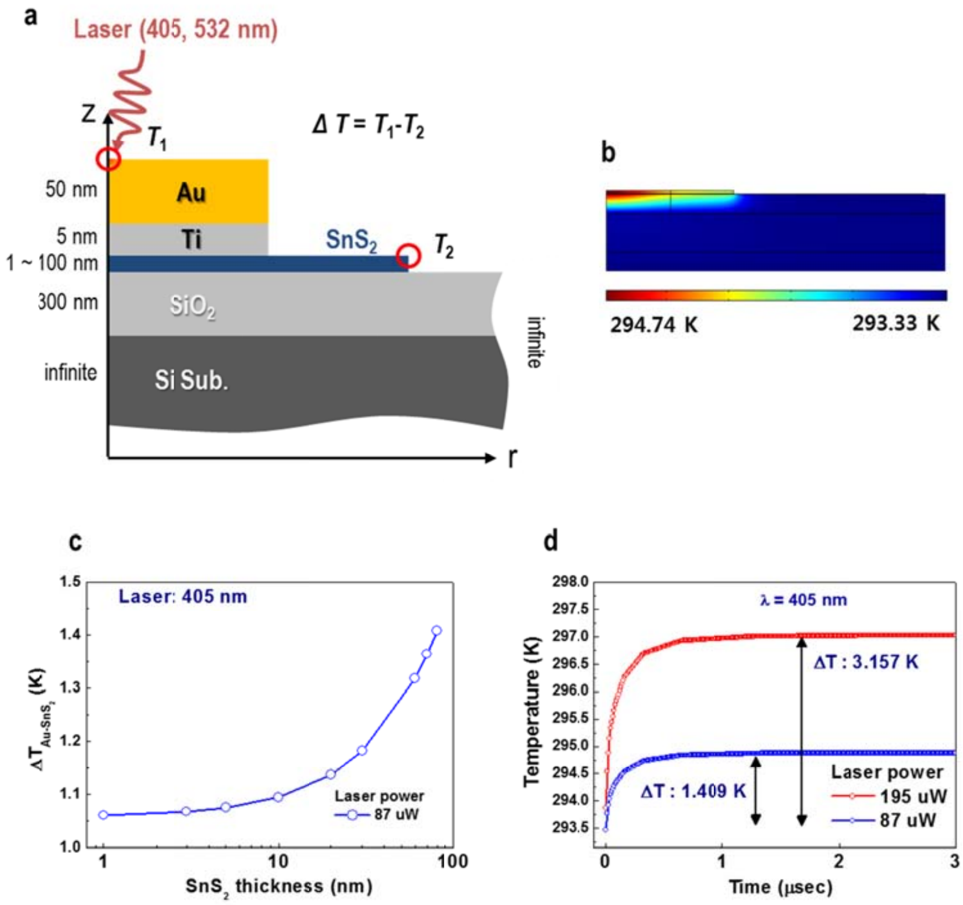

Supplementary Figure 13. Temperature difference simulation used for Seebeck coefficient calculation. (**a**) A modeled system for the ΔT estimation, induced from the focal laser heating in our study. (**b**) Two-dimensional temperature distribution at 1 μsec after illumination (optical power = 87 μW, λ = 405 nm). (**c**) Simulated ΔT saturation dependence on SnS_2 film thickness. (**d**) Temperature saturation occurs at 1 μsec for 120 nm sample.

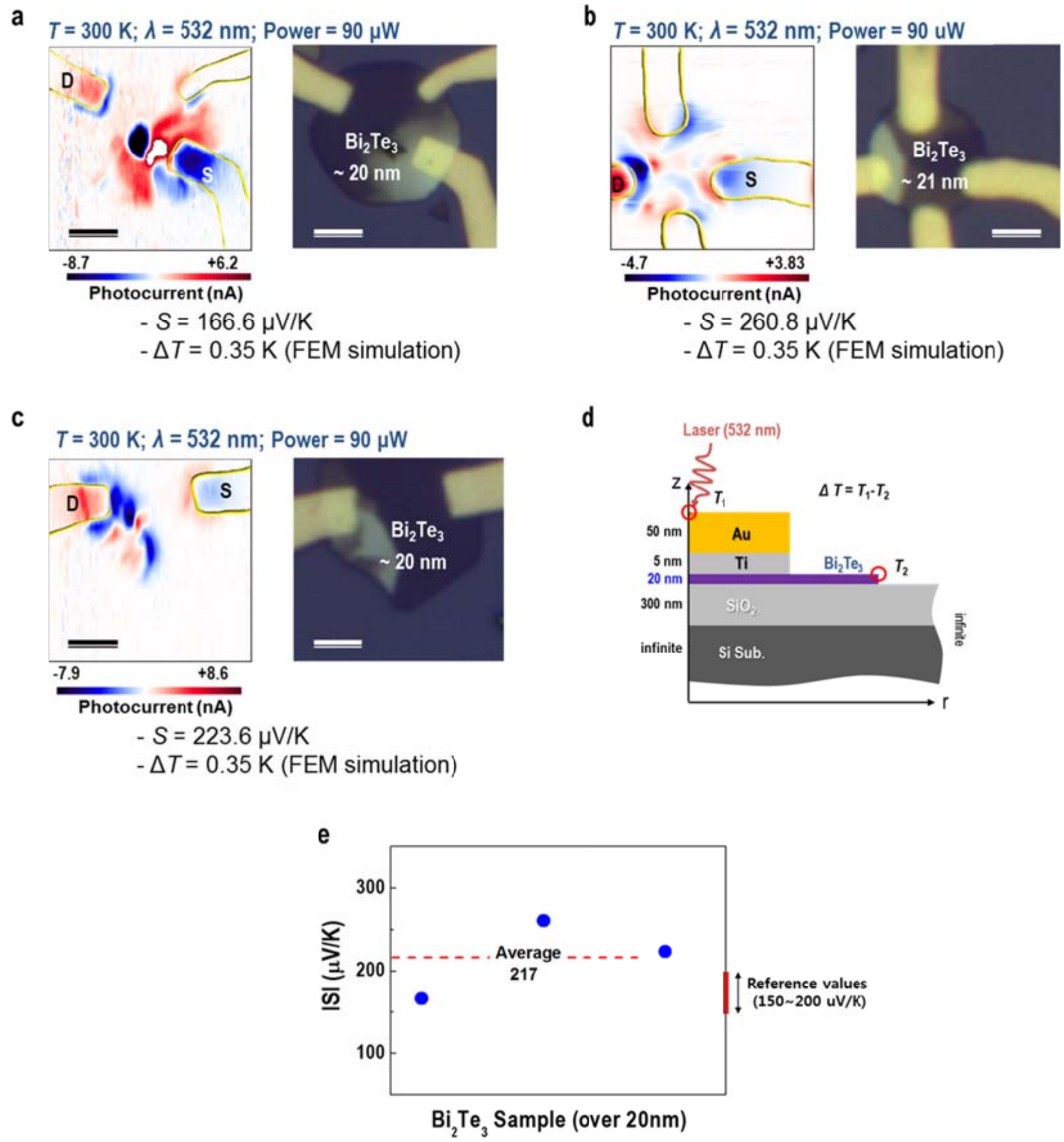

Supplementary Figure 14. (**a-c**) Photocurret map and optical microscopy images, Seebeck coefficients and values of temperature difference in Bi_2Te_3 samples. Scale bar, 4 μm (**a**), 3 μm (**b**) and 2.5 μm (**c**). (**d**) Structure for ΔT estimation using Finite Element Method (FEM). (**e**) Obtained Seebeck coefficient average (217 μV K^{-1}) calibration samples compared to previously reported values (150~200 μV K^{-1}). When compared to previously reported values in **Supplementary References 5–7**, we found a calibration factor of 1.09-1.45.

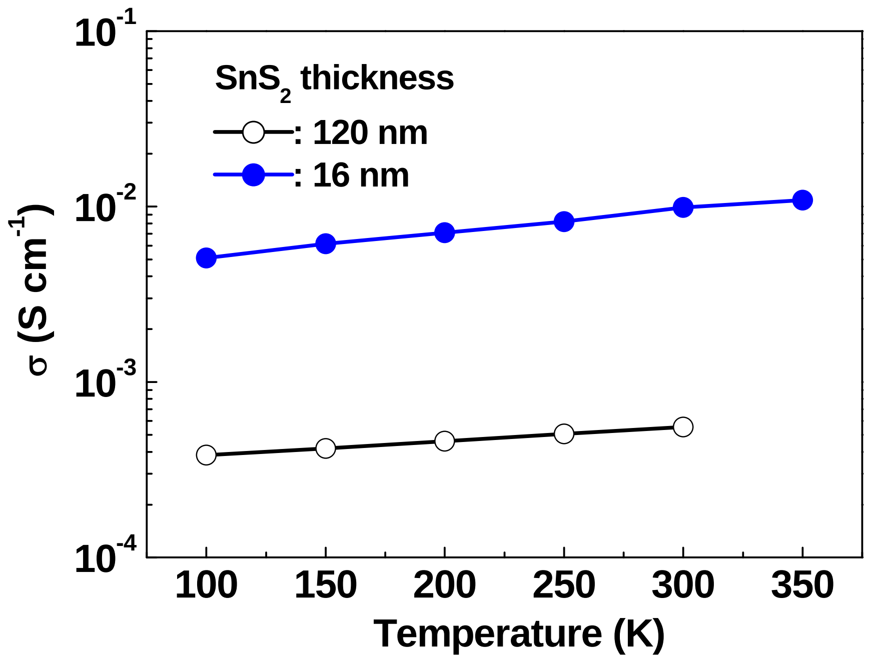

Supplementary Figure 15. Temperature dependent Electrical conductivity measurements for 16 and 100 nm samples using techniques shown in Supplementary Fig. 11.

Supplementary Tables

Supplementary Table 1.

List of material properties values

	**Au**	**Ti**	**SnS_2**	**SiO_2**	**Si**
d (nm)	50	5	20	300	inf
ρ (kg m^{-3})	19300	4507	4500	2650	2330
C (J kg^{-1} K^{-1})	129	523	355	1000	710
κ (W m^{-1} K^{-1})	315	21.9	0.85	1.38	149
α (cm^{-1}) @ 532 nm	568670	597860	2000	0.024	12233
α (cm^{-1}) @ 405 nm	607140	669350	100000	0.024	106140

Supplementary Notes

Supplementary Note 1

To find the mechanism about the physical origin of this abnormal change of electrical conductivity, we have performed a computational calculation using the following assumptions:

It is known that sulfur (S) vacancies exist in SnS_2 and MoS_2 which are the semiconductor of the type MS_2, with M a metal atom (Sn, Mo, etc.) and a S chalcogen atom resulting in the n-type behavior of SnS_2 and MoS_2. The S vacancies on the surface tend to form covalent bonds with sulfur-containing groups and electronic states in the band gap[1,2]. In addition, Burton, L. A. *et al.* showed that the S vacancy defect formation energy of 1.8 eV and concentrations (donor type) of 2.21×10^{12} cm^{-3} in SnS_2 wear calculated by using a computation of the DFT level of electronic structure methods[3]. We attempted a TCAD simulation using the above as reference for an assumed sample of shape shown in **Supplementary Fig. 7a**. Our TCAD results in **Supplementary Fig. 7b** showed that for reduced SnS_2 thickness, the total electron density is affected more proportionately by surface doping. That is, of course that thinner samples will have total electron density more affected by surface doping than thicker ones. A simple illustration showing this effect is shown in **Supplementary Fig. 7d**. The *I-V* simulations based of the given electron densities are shown in **Supplementary Fig. 7c**, showing an increase in conductivity for the thinner samples. Meanwhile just this effect does not completely explain the larger changes observed in experimental data. We believe that this simple model cannot include all the factors (surface chemical modification such as incorporation of hydrogen, water, dangling bonds and defect engineering[4]) which result in the final conductivity values.

Supplementary Note 2

Laser-induced temperature estimation

To calculate the Seebeck coefficient through expression $S = V_{oc}/\Delta T$, it is necessary to estimate the temperature difference (ΔT) between the Au electrode and the SnS_2 flake induced by the laser heating (V_{oc} is open circuit voltage). The heat exchange process was simulated using a commercially available Finite Element Method (FEM) software

(COMSOL Multiphysics) as shown in **Supplementary Fig. 13**. The system is modelled as presented in **Supplementary Fig. 7a**: a 2D model representative of the cross section of the single-layer SnS_2 FET which is symmetric around the axis of the incident laser beam (z axis). This solution allows to increase the number of mesh elements, yielding a more precise solution, while ensuring a reasonable solution time. Note that the roughness of the electrode, as determined with Atomic Force Microscopy is around 5 nm, much smaller than the excitation wavelength, and therefore does not play a role in the laser absorption by the electrodes. The material properties used for the simulation are listed in Supplementary Table 1. The incident laser power density is calculated from the measured power assuming a diffraction limited spot, a 68% transmission through the objective and the absorption through the thickness of the material according to

$$I = I_0(1 - \exp(-\alpha d))$$

where α is the absorption coefficient of the Au/Ti electrode and d is its thickness. We assume that all the incident laser power is converted into heat. The boundary conditions are as follows: symmetry around the z axis (r = 0), incoming heat flux density on the area of the spot size equal to the optical power density calculated above, free heat outlet for the SiO_2 and Si free boundaries (as their in-plane size is much bigger than the one of SnS_2 and electrodes), room temperature fixed at the bottom of the silicon chip. This is reasonable since the bottom is in good thermal contact with the chip carrier and the cryostat that can be modelled as a thermal bath at constant temperature due to the much bigger mass than the measured sample. Radiation from the other surfaces is neglected.

Supplementary Note 3

ΔT calibration using Bi_2Te_3

Our experimental and FEM simulation methods for three different Bi_2Te_3 samples (20 nm, 21 nm, 20 nm) which we expected to approach near bulk values. The Seebeck coefficient of Bi_2Te_3 we extracted for these samples was 166.6 μV K^{-1}, 260.8 μV K^{-1}, and 223.6 μV K^{-1} respectively. We plotted the obtained Seebeck coefficient values along with the range of reported reference values as shown in **Supplementary Fig. 14e**. We found an overestimation of the average Seebeck Coefficient of 1.09~1.45 when compared to previously reported values (150-200 μV K^{-1}) in **Supplementary References 5-7**.

Supplementary References

1. Cho, K. *et al*. Electrical and optical characterization of MoS_2 with sulfur vacancy passivation by treatment with alkanethiol molecules. *ACS Nano* **9**, 8044-8053 (2015).

2. Kodama, N. *et al*. Electronic states of sulfur vacancies formed on a MoS2 surface. *Jpn. J. Appl. Phys*. **49**, 08LB01 (2010).

3. Burton, L. A. *et al*. Synthesis, characterization, and electronic structure of single crystal SnS, Sn_2S_3, and SnS_2. *Chem. Mater.* **25**, 4908−4916 (2013).

4. Guo, Y. *et al*. Surface chemical-modification for engineering the intrinsic physical properties of inorganic two-dimensional nanomaterials. *Chem. Soc. Rev.* **44**, 637-646 (2015).

5. Li, A. H. *et al*. Electronic structure and thermoelectric properties of Bi_2TeS_3 crystals and grapheme-doped Bi_2TeS_3. *Thin Solid Films* **518** (24), 358−363 (2010).

6. Tan, J. *et al*. Thermoelectric properties of bismuth telluride thin films deposited by radio frequency magnetron sputtering. *Proc. SPIE* **5836**, Smart Sensors, Actuators, and MEMS II, 711 (2005).

7. Eibl, O., Nielsch, K., Peranio, N., Völklein, F., *Thermoelectric Bi_2Te_3 Nanomaterials* (Wiley-VCH Verlag GmBh & Co. KGaA, Weinheim, Germany, 2015).
